# Relationships between tilt angles of rectus muscles and positions of rectus muscle pulleys in patients with sagging eye syndrome

**DOI:** 10.1007/s10384-024-01141-8

**Published:** 2025-02-04

**Authors:** Reika Kono, Ichiro Hamasaki, Fumiko Kishimoto, Kiyo Shibata, Shin Morisawa, Yuki Morizane

**Affiliations:** 1 Department of Ophthalmology, Okayama University Graduate School of Medicine, Dentistry, and Pharmaceutical Sciences, 2-5-1 Shikata-cho;Kita-ku, Okayama City, Okayama 700-8558 Japan; 2 Division of Ophthalmology, Ibara City Hospital, Ibara City, Okayama Japan

**Keywords:** Orbital pulley, Sagging eye syndrome, Distance esotropia, Cyclovertical strabismus, Aging

## Abstract

**Purpose:**

To examine the relationship between the rectus muscle (RM) angle and RM pulley displacement in patients with sagging eye syndrome (SES) without myopia.

**Study design:**

Retrospective cross-sectional case series.

**Methods:**

High-resolution quasi-coronal magnetic resonance imaging (MRI) data from 20 orbits of ten Japanese patients with SES but without high myopia were analyzed. The patients had no abduction deficiency. The RM angles were measured between the major axes of the horizontal and vertical RMs relative to the vertical and horizontal planes, respectively. The positions of the RM pulleys relative to the center of the globe were analyzed as previously described.

**Results:**

The mean age of the patients was 75.8 ± 4.5 years (standard deviation). The average axial length was 23.6 ± 0.6 mm. The lateral rectus (LR) muscle angle (22 ± 6°) had moderate negative correlations with the inferior displacement of the inferior rectus (IR), superior rectus (SR), and LR pulleys (r =– 0.63,– 0.45, and– 0.45, respectively); however, no change was observed in the medial rectus (MR) pulley (r =– 0.41). No correlations were found between the angles of the SR (4 ± 8°), IR (– 13 ± 8°), and MR (– 1 ± 6°) muscles and the positions of the RM pulleys.

**Conclusion:**

Given the correlation between increased LR muscle angle and inferior displacement of adjacent RM pulleys in SES, the LR muscle angle may serve as a diagnostic clue, even when inferior displacement is not identifiable on MRI. Further confirmation in larger studies is warranted.

## Introduction

Binocular misalignment causes approximately two-thirds of new-onset diplopia cases in adults aged > 40 years in California [1] and one-third in older patients in Japan [2]. The two most common causes of adult diplopia are sagging eye syndrome (SES; 31% of cases) and superior oblique (SO) muscle palsy (10% of cases) [1]. Based on these reports, SES, a frequent cause of acquired diplopia in adults, has received considerable attention in recent years. SES was investigated by Demer et al. [3–5]. In middle-aged or older adults with distance diplopia as their primary symptom, factors such as distance esotropia (DE) or cyclovertical strabismus (CVS) often coincide, leading to issues like inferior lateral rectus (LR) pulley displacement, thinning, or elongation of the lateral rectus-superior rectus band (LR-SR band), greater vertical angulation of the LR as seen in magnetic resonance imaging (MRI), as well as the presence of superior sulcus deformity, aponeurotic blepharoptosis, and a baggy eyelid in the adnexa. The inferior displacement of the LR pulley is the basis of SES diagnosis [4, 5]. These clinical observations and orbital MRI findings suggest that the pathophysiology of SES is an age-related change [3, 4]. In contrast, the displacement of the LR is also observed in highly myopic eyes with strabismus, termed heavy eye syndrome (HES) [3, 6, 7]. This phenomenon is presumably due to mechanical downward pressure on the LR, as the posterior part of the globe exits the muscular cone due to dilation of the posterior part of the globe in the highly myopic eye [6]. SES and HES have characteristic findings and can be differentially diagnosed by orbital MRI. Chaudhuri et al. [4] included high myopia as a criterion for SES unless there was significant myopic degeneration, and there are reports on the characteristics of SES in individuals with high myopia [7, 8].

The inferior displacement of the LR pulley, a hallmark of SES, was first reported by Rutar [3] and can be used to diagnose SES. Masquerading SO palsy (MSOP) without SO atrophy sometimes resembles the clinical findings of CVS-type SES [5]. MSOP is considered if the positions of the LR pulley do not differ between hypertropia and hypotropia on MRI [5]. CVS-type SES is considered if the inferior shift of the LR pulley is greater in the eye with hypotropia than in the eye with hypertropia [4, 5]. Thus, cases exist wherein differential diagnoses can be made by evaluating the inferior shift of the LR pulley. This evaluation is of significant diagnostic importance. However, determining the extent of the inferior shift of the LR pulley in coronal MRI images can be challenging, depending on the alignment of the eye’s visual axis with the imaging plane during the scan. In such cases, seeking indirect indicators to assess the LR pulley’s inferior shift can be beneficial. One example is the tilt angle of the LR. The LR angle (the angle between the long axis of the LR and the perpendicular line) increases significantly with an inferior shift of the LR pulley in Japanese patients with age-related DE and high myopia, and, on MRI the LR angle can be useful in determining the degree of inferior shift of the LR pulley [8]. An increased temporal tilt of the superior LR, i.e., the LR angle, is also a characteristic SES finding [4].

Symmetrical LR sag is associated with divergence paralysis esotropia and asymmetrical LR sag of >1 mm with cyclovertical strabismus in patients with SES [4]. A histological study shows that the LR-SR band, global side of the LR pulley, and inferior oblique (IO) pulley that connects to the global side of the LR pulley indicate a marked decrease in collagen content in healthy older adults [9], presumed to be one cause of inferior shift of the LR pulley. In contrast, the collagen content was relatively maintained in the MR pulley, MR-IR band (the band of connective tissue connecting the MR and IR pulleys), and IR pulley, compared to the LR-SR band, even in older adults [9]. These findings suggest that the effects of aging on connective tissue are expected to differ in each rectus muscle (RM) pulley depending on the histological and anatomical characteristics of each RM pulley. While some studies report abnormal RM positioning other than the LR [3–5, 10, 11] in SES, few studies [8] have thoroughly examined the relationship between the degree of inferior shift of the LR pulley position or increased LR tilt angle, which appears as an age-related change, and those of other RMs. We hypothesized that age-related changes in the LR pulleys of SES specifically affect the IR and SR pulleys adjacent to the LR pulley, while the MR pulley would be less affected, and that the inferior displacement of the LR pulley and increased LR tilt angle, findings of age-related changes, would correlate with the positions of the IR and SR pulleys.

## Subjects and methods

This retrospective clinical case series was based on medical records. This study complied with the Declaration of Helsinki and the Ethical Guidelines for Medical Research Involving Human Subjects. The requirement for informed consent was waived since the analysis used deidentified clinical data, collected after the receipt of each patient’s consent to treatment. Furthermore, we employed an opt-out approach to secure consent for this study, facilitated by a poster and website. The study design was approved by the Ethics Committee of Okayama University Hospital (No. K1507-021).

The study included 20 orbits of ten patients diagnosed with SES without high myopia based on clinical and orbital MRI findings during medical examinations for strabismus conducted between 2010 and 2022 at Okayama University Hospital and affiliated hospitals. Although highly myopic eyes in cases without signs of a “heavy eye” syndrome do not preclude the diagnosis of SES [4, 7, 8], we excluded these eyes to eliminate the unknown effects of high myopia on the pathogenesis of SES, focusing solely on SES without high myopia.

Ocular misalignment was measured for the primary gaze position at both distance (5 m) and near (0.3 m) using the alternate prism cover test. Subjective cyclotorsion was examined using the Double Maddox rod test.

### Definition of SES in this study

The diagnosis was based on clinical and MRI findings, in accordance with Wei et al. [5]. Clinical findings included patients aged > 40 years who experienced acquired distance diplopia (with preserved binocular single vision at near distance), DE (10 ∆ or less esophoria or orthophoria at near distance), or CVS (subjective extorsion of the inferior strabismus eye not less than that of the superior eye). The patients exhibited no abduction limitations and had normal horizontal saccades. MRI findings included inferior displacement of the LR pulley in at least one eye, with no evidence of SO atrophy on orbital MRI. The presence of inferior displacement of the LR pulley was determined by comparing the pulley positions with those previously reported in 14 eyes of young healthy Japanese adult participants without high myopia (mean ± standard deviation [SD] age: 30.6 ± 2.2 years, range 27–32 years) [12]. The inferior vertical position of the LR pulley was defined as the inferior displacement based on the 95% confidence interval (– 1.99 ± 0.52 mm) reported for healthy young participants. Patients with LR pulley inferior displacement in one or both eyes were considered to have LR pulley inferior displacement. Patients with high myopia (ocular axis > 26 mm or– 6.0 D), history of trauma or strabismus surgery, central vascular disease, central nervous system disorder, cranial nerve palsy, thyroid eye disease, myasthenia gravis, childhood-onset strabismus, or diseases that were obvious causes of diplopia or strabismus were excluded.

### Imaging methods and conditions in orbital MRI

**Fig. 1:**
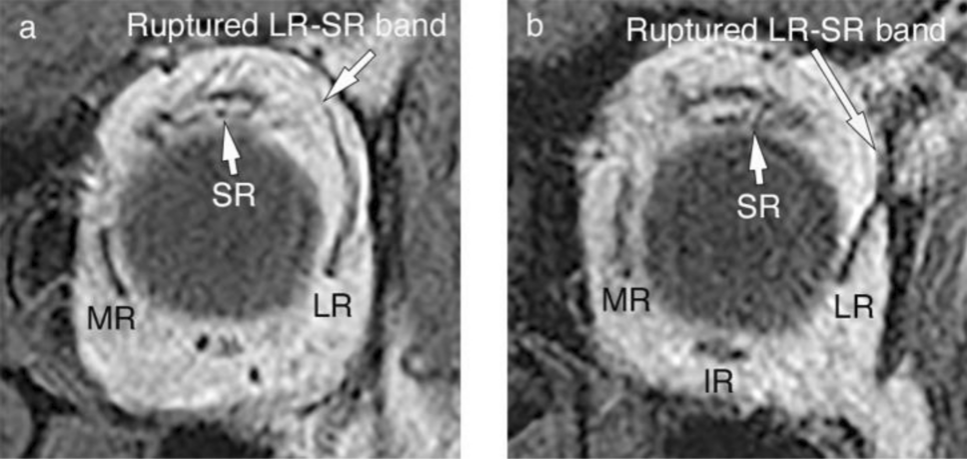
T1-weighted quasi-coronal images of patients with sagging eye syndrome. **a** Moderate rupture of the lateral rectus (LR)- superior rectus (SR) band with marked LR and medial rectus (MR) muscle sags in the left orbit of patient number 10. **b** Severe rupture of the LR-SR band in the left orbit of patient number 2. *IR* inferior rectus

**Fig. 2:**
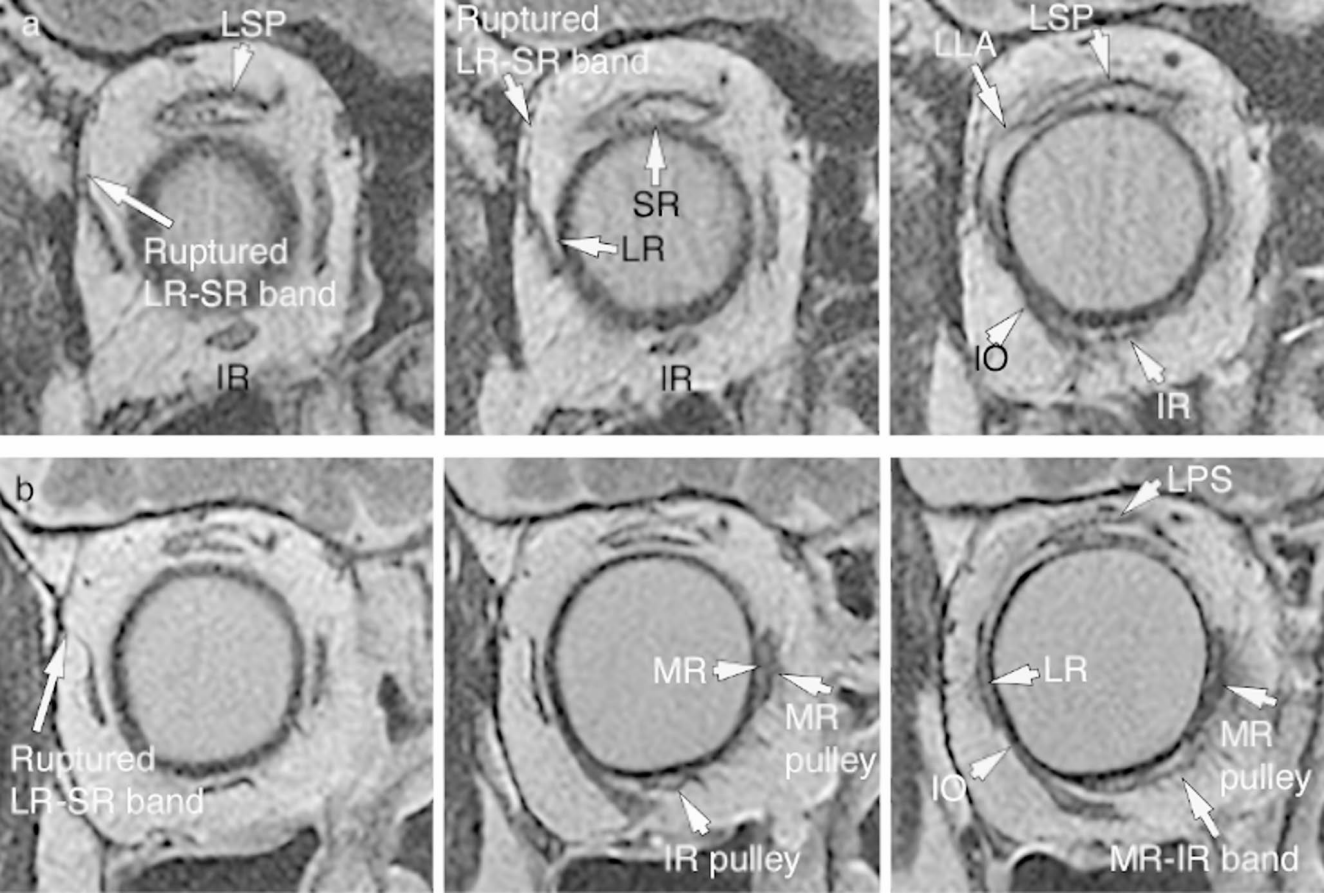
T2-weighted quasi-coronal images of patients with sagging eye syndrome. **a** Left orbit of patient number 3 (80-year-old female). The image in the left column was 2 mm anterior to the globe/optic nerve junction, and that in the center column was 4 mm anterior. The tilt of the LR with a ruptured LR-SR band (28°) was greater than that in patient number 4 (13°). The pulley positions of the MR (–2.5 mm) and IR (–14.5 mm) were inferior to those of patient number 4 (–0.1 mm and –9.8 mm, respectively). The LLA bulged superotemporally in the image of the right column (6 mm anterior to the globe/optic nerve junction). **b** Right orbit of patient number 4 (75-year-old female). A ruptured LR-SR band was observed in the left column (4 mm anterior to the globe-optic nerve junction). In the center (6 mm anterior) and right column (8 mm anterior), the IR pulley orbital, MR pulley orbital, and MR-IR band had low signal intensities. The MR and IR, which had higher signal intensities than the pulley, were surrounded by a lower signal pulley. Compared with patient number 3, no inferior shifts were observed in the MR and IR pulleys. The connection between the global side of the LR and the orbital side of the IO was observed. *LR* lateral rectus, *MR* medial rectus, *SR* superior rectus, *IR* inferior rectus, *LLA* lateral levator aponeurosis, *LPS* levator palpebrae superioris, *IO* inferior oblique

MRI was performed using a Signa Excite 3T scanner (GE Healthcare) following a previously published method [8]. The conditions for quasi-coronal T1-weighted imaging were: matrix, 256 × 256; field of view, 12 cm; slice thickness, 3 mm; and repetition time/echo time (TR/TE), 750/11.1–11.7 ms (Fig. 1). The conditions for quasi-coronal T2-weighted imaging were: matrix, 256 × 256; field of view, 12 cm; slice thickness, 2 mm; and TR/TE, 4000/92 ms (Fig. 2).

T1 quasi-coronal MRI images at approximately 9 mm posterior to the center of the eyeball were acquired to measure the pulley positions and the angulations using ImageJ (https://imagej.nih.gov/ij/).

### Positions of the RM pulleys

The positions of the RM pulleys were analyzed using a normalized, oculocentric coordinate system based on their horizontal and vertical coordinates relative to the center of the globe at position “0,” as described by Clark and Demer [13]. The left orbital image was analyzed after being flipped horizontally. The temporal positions of the RM pulleys were indicated by their positive and nasal positions using negative horizontal coordinates relative to the center of the globe. The superior positions of the RM pulleys were indicated by their positive and inferior positions using negative vertical coordinates.

### Tilt angles of the RMs

**Fig. 3:**
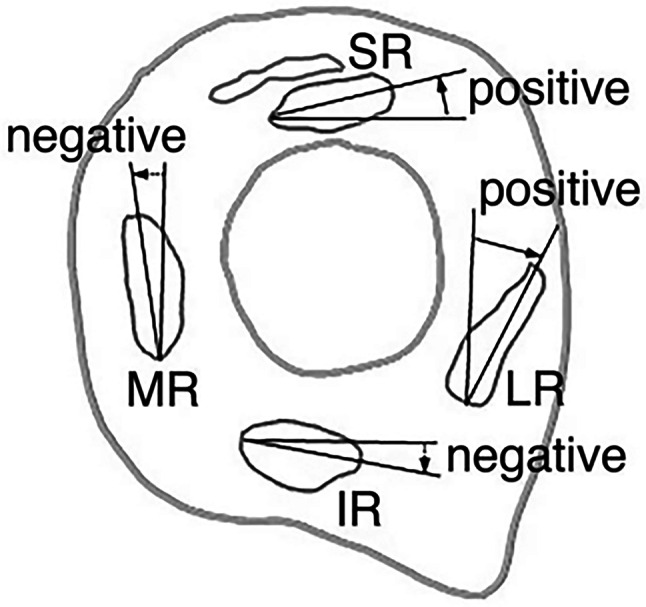
Schema of angles of the rectus muscles in the right orbit. A positive value was defined when the upper major axis of the medial rectus (MR) or lateral rectus (LR) muscle was temporally tilted compared to the perpendicular line or when the superior rectus (SR) or inferior rectus (IR) muscle major axes were superiorly tilted compared to the horizontal line

The angle of the long axis of the RM cross-section was measured at the location of the rectus pulley using ImageJ. The ellipse that exhibited the best fit to the cross-sectional area of the RM was obtained. The length of the major axis of the best-fit ellipse was computed. The angle between the major axis of the RM and a line running perpendicularly or horizontally was measured to obtain the RM angle (Fig. 3) [4, 8]. The ellipse that exhibited the best fit to the cross-sectional area of the RM was computed. The angle between the major axis of the horizontal RM and a line running parallel to the y-axis of the image yielded the LR and medial rectus (MR) angles. The angle between the major axis of the horizontal RM and the line parallel to the x-axis of the image (horizontal) provided the SR and inferior rectus (IR) angles (Fig. 3). The left orbital image was analyzed after being flipped horizontally.

### LR-SR band

Qualitative morphological evaluation of the LR-SR bands was performed using quasi-coronal T1-weighted MRI images [8]. The LR-SR band was assessed for thinning, elongation, displacement, or rupture according to the criteria of previous reports [8], if it did not retain a normal structure along the sclera of the eye (Fig. 1).

### Statistical analyses

The positions of the RM pulleys were compared to those of young healthy participants [12] using Wilcoxon rank sum test with continuity correction. Moreover, statistical analyses were performed to examine the relationships between the vertical positions of the LR pulley or the angles of the LR muscle and the positions of the RM pulleys using Spearman’s rank correlation. Correlations between the vertical positions of the LR pulley or the angles of the LR muscle and the angles of the MR, SR, or IR muscles were also assessed. EZR, which is a free statistical software (https://www.jichi.ac.jp/saitama-sct/SaitamaHP.files/statmed.html), was used for statistical analyses [14]. Statistical significance was set at P < 0.05.

## Results

The clinical findings of the 20 eyes of ten patients with SES are presented in Table 1. Two men and eight women were included (mean ± SD age: 75.8 ± 4.5 years, range 68–82 years), 80% of whom had vertical deviations (two patients with DE, three with CVS, and five with DE and CVS). The average axial length was 23.6 ± 0.6 mm, and the mean equivalent spherical value of the 12 eyes (excluding the two pseudophakic eyes and six eyes with undescribed data) was 0.03 ± 1.39 diopters. The mean exocyclotropia in seven patients (excluding three patients with undescribed data) was 4.3 ± 3.2°. All 20 examined orbits displayed ruptured LR-SR bands, with six eyes showing mild to moderate rupture and 14 eyes exhibiting severe rupture of the LR-SR band (Fig. 1). Three patients were treated with prism glasses without surgery, three patients underwent strabismus surgery exclusively, two patients received prism glasses following strabismus surgery, and for two patients, the treatment method was unknown.

**Table 1:** Clinical data of patients with sagging eye syndrome

Patients	Sex	Age (years)	Duration of diplopia (years)	Ocular position (∆) at distance at near
1	Male	68	3	LH3 4X’
2	Female	76	1.5	LHT6 8X’ LH(T)’6
3	Female	80	0.5	14ET LHT5 LH(T)’4
4	Female	82	1	12ET RHT5 4X’ RHP’5
5	Female	77	5	12ET LHT8 LH(T)’5
6	Female	73	2	6ET 2X’
7	Female	69	4	LH(T)4 4X’ LH(T)’4
8	Male	77	2.5	10ET LHT6 LH(T)’3
9	Female	77	1	4E LHT3 4X’ LH’4
10	Female	79	10	18ET 4E(T)’

*E* esophoria, *ET* esotropia, *LH* left hyperphoria, *LHT* left hypertropia, *RH* right hyperphoria, *RHT* right hypertropia, *X* exophoria

### Positions of the RM pulleys

**Fig. 4:**
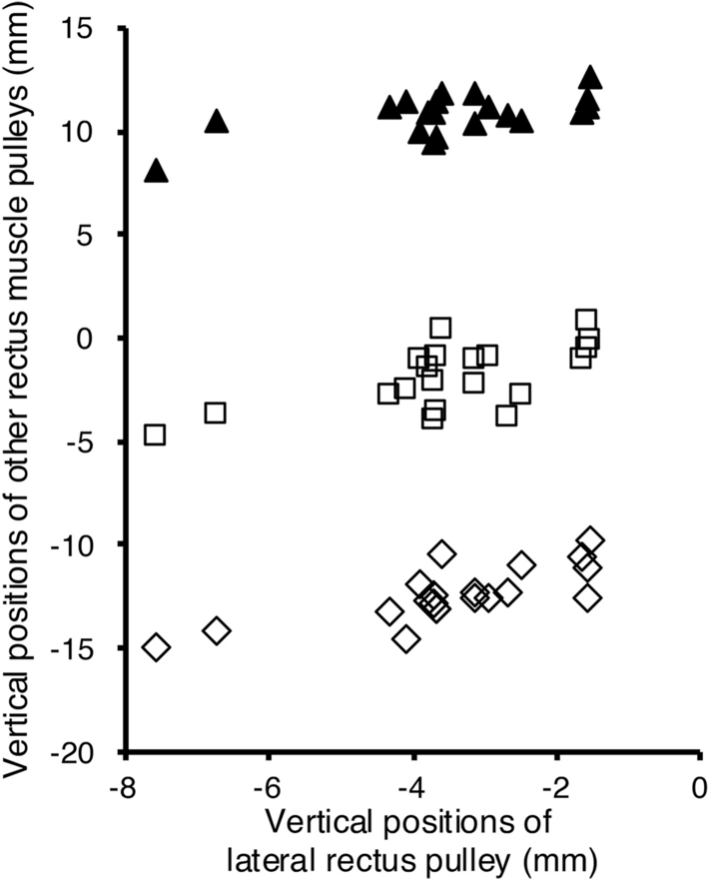
Relationships between the vertical positions of the lateral rectus pulley and the vertical positions of the three rectus muscle pulleys. The inferior displacements of the inferior rectus (IR) (Spearman's rank correlation, r = 0.74, P < 0.001), medial rectus (MR) (r = 0.59, P < 0.01), and superior rectus (SR) pulleys (r = 0.46, P < 0.05) increased when those of the lateral rectus (LR) pulley increased. (Square) MR pulley, (triangle) SR pulley, (diamond) IR pulley. The vertical positions of the rectus muscle pulleys are indicated by coordinates relative to the center of the globe at position “0”.

The mean (± SD) positions of the RM pulleys relative to the center of the globe are listed in Table 2. Compared to young healthy participants [12], the LR and SR pulleys of patients with SES exhibited significant inferior displacements (P < 0.01), and the SR pulleys of patients with SES exhibited significant temporal displacements (P = 0.04). The inferior displacements of the IR, MR, and SR pulleys increased when those of the LR pulley increased (Spearman’s rank correlation, r = 0.74; P < 0.001, r = 0.59; P < 0.01, and r = 0.46; P < 0.05, respectively; Fig. 4, Table 3). No correlations were found between the vertical and horizontal positions of the LR and RM pulleys.

**Table 2:** Positions of the rectus muscle pulleys

Group	Positions of the rectus muscle pulley: mean ± SD (mm)							
	LR pulley		SR pulley		IR pulley		MR pulley	
	Horizontal	Vertical	Horizontal	Vertical	Horizontal	Vertical	Horizontal	Vertical
SES	10.15 ± 0.59	– 3.46 ± 1.54	– 0.90 ± 0.81	10.83 ± 0.97	– 4.21 ± 1.10	– 12.38 ± 1.32	– 12.71 ± 0.78	– 1.85 ± 1.57
Young healthy participants [11]	10.16 ± 0.84	– 1.99 ± 1.07	– 1.56 ± 0.82	10.65 ± 0.86	– 4.48 ± 0.98	– 12.33 ± 0.84	– 13.22 ± 0.74	– 1.11 ± 0.98
*P*-value*	0.77	< 0.01	0.04	0.36	0.62	0.82	0.10	0.18

*LR* lateral rectus, *SR* superior rectus, *IR* inferior rectus, *MR* medial rectus, *SD* standard deviation, *SES* patients with sagging eye syndrome

*Wilcoxon rank sum test with continuity correction

**Table 3:** Angles of the rectus muscles in patients with sagging eye syndrome

Rectus muscles	Angles mean ± SD (°)
Lateral rectus muscle	22 ± 6
Superior rectus muscle	4 ± 8
Inferior rectus muscle	– 13 ± 8
Medial rectus muscle	– 1 ± 6

*SD* standard deviation

### Tilt angles of the RMs

**Fig. 5:**
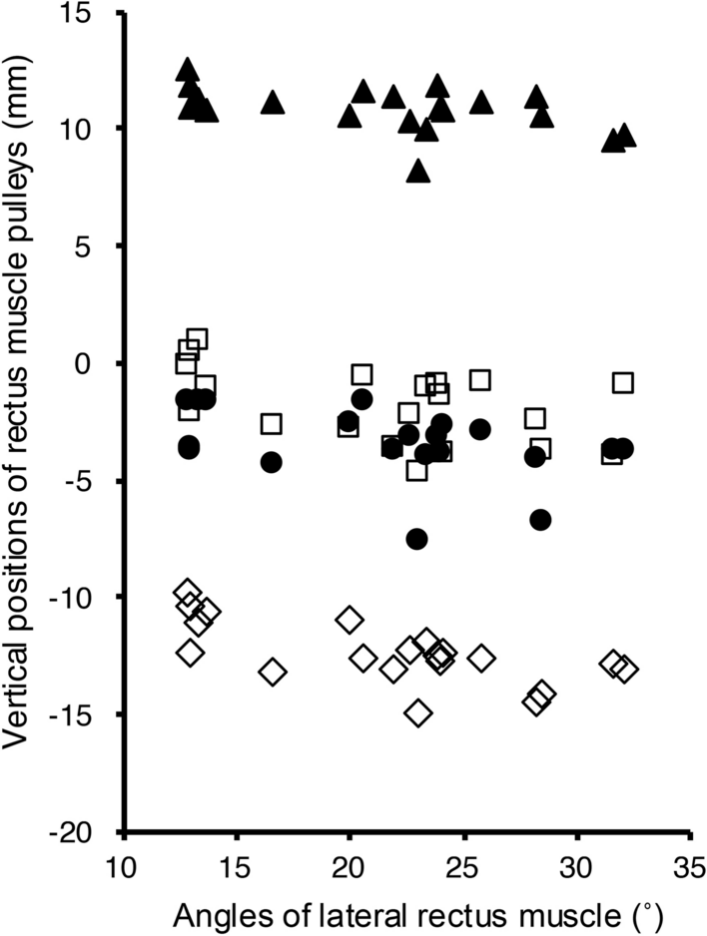
Relationships between the lateral recuts angle and the vertical positions of the rectus muscle pulleys. The lateral rectus (LR) angle increased significantly with the inferior displacement of the inferior rectus (IR) (Spearman's rank correlation, r =– 0.63, P < 0.01), superior rectus (SR) (r =– 0.45, P < 0.05), and LR pulley (r =– 0.45, P < 0.05); however, not with medial rectus (MR) pulleys (r =– 0.41, P = 0.07). (Square) MR pulley, (triangle) SR pulley, (circle) LR pulley, (diamond) IR pulley. The vertical positions of the rectus muscle pulleys are indicated by coordinates relative to the center of the globe at position “0”.

The mean (± SD) angles of the RM muscles are presented in Table 3. The angle of the LR had moderate negative correlations with the inferior displacement of the IR, SR, and LR pulleys (r =– 0.63; P < 0.01, r =– 0.45; P < 0.05, and r =– 0.45; P < 0.05, respectively), but not with the MR pulley (P = 0.07; Fig. 5, Table 4). No correlations were found between the position of the LR pulley or the angle of the LR muscle and the angles of the SR, IR, or MR muscles (Table 5).

**Table 4:** Relationships between the vertical position of the lateral rectus pulley or the angle of the lateral rectus muscle and the positions of the rectus muscle pulleys

	Vertical positions of the pulley				Horizontal positions of the pulley			
	LR	SR	IR	MR	LR	SR	IR	MR
Vertical position of the LR pulley		r = 0.46	r = 0.74	r = 0.59	r = 0.11	r =–0.41	r = 0.01	r = 0.23
		P < 0.05	P < 0.001	P < 0.01	P = 0.65	P = 0.07	P = 0.98	P = 0.33
Angle of the LR muscle	r =– 0.45	r =– 0.45	r =– 0.63	r =– 0.41	r =– 0.16	r =– 0.05	r = 0.29	r =– 0.26
	P < 0.05	P < 0.05	P < 0.01	P = 0.07	P = 0.51	P = 0.85	P = 0.21	P = 0.27

Statistical analysis was performed using Spearman’s rank correlation. r represents the correlation coefficient. P represents the probability value

*LR* lateral rectus, *SR* superior rectus, *IR* inferior rectus, *MR* medial rectus

**Table 5:** Relationships between the vertical position of the lateral rectus pulley or the angle of the lateral rectus muscle and the angles of other rectus muscles

	Angles of the rectus muscles		
	Superior rectus muscle	Inferior rectus muscle	Medial rectus muscle
Vertical position of the lateral rectus pulley	r = 0.03	r =– 0.13	r =–0.34
	P = 0.91	P = 0.58	P = 0.15
Angle of the lateral rectus muscle	r =– 0.03	r =– 0.02	r = 0.13
	P = 0.91	P = 0.95	P = 0.57

Statistical analysis was performed using Spearman’s rank correlation. r represents the correlation coefficient. P represents the probability value

## Discussion

In the present study, compared to healthy young healthy participants, those with SES with full abduction and no myopia showed inferior displacement of the LR pulley and temporal displacement of the SR pulley (Table 2). In contrast, previous studies [4, 5] utilizing methods similar to ours for measuring pulley positions report inferior and temporal displacement of the LR and IR pulleys in patients with SES compared to those in young healthy individuals (average age: 28.5 years, range 21–33 years). Factors that may have contributed to the different results include the inclusion or exclusion of high myopia, variations in the participant selection methods, and potential bias introduced by the analyst when tracing the images. Chaudhuri et al. [4] report that patients with SES exhibited thinning, elongation, or frequent rupture of the LR-SR band, with ruptured LR-SR bands observed in approximately 80% of cases. In the current study, ruptured LR-SR bands were observed in all patients. In summary, given that inferior displacement of the LR pulleys and degeneration of the LR-SR band were common MRI findings in patients with SES in both the current study and previous reports, these findings warrant special attention in imaging diagnosis.

As hypothesized, the angle of the LR muscle and the inferior displacement of the IR, SR, and LR pulleys were moderately correlated, but not with the MR pulley. In contrast, the inferior displacement of the LR pulley was correlated not only with the inferior displacement of the IR and SR pulleys but also with the MR pulleys; however, it did not correlate with the horizontal displacements of the four RM pulleys.

In this study, among the patients with SES who met the criteria for the inferior displacement of the LR and degenerated LR-SR band, the correlations between the inferior displacement of the LR and the inferior displacement of the IR, MR, and SR pulleys were more pronounced in that sequence. As mentioned above, the inferior displacement of the LR pulley is one of the hallmarks imaging findings in SES; however, the inferior displacement of MR and IR pulleys varies according to the type of SES, strabismic eye, and previous reports [3–5, 10, 11]. In patients with SES, the correlation between the inferior displacement of the LR pulley and that of the other RM pulleys suggests that age-related connective tissue alterations could affect all four RM pulleys.

The angle of the LR muscle was correlated with the vertical displacement of the LR, IR, and SR pulleys but not with the MR pulley. Histological studies of pulleys report that the MR pulley, MR-IR band, and IR pulley have more elastin and smooth muscle cells and greater collagen content than the LR-SR band [9, 15]. Moreover, similar to the LR-SR band, the MR pulley, MR-IR band, and IR pulley also show a decrease in collagen content and thickness with age and retain more collagen content than the LR-SR band [9]. Owing to these histological characteristics, the pulley structures around the MR pulley are more easily preserved than those around the LR pulley, even in old age, suggesting that the LR and surrounding pulley structures are more vulnerable to age-related changes.

The mean (± SD) angulation of the LR with respect to the vertical is reported to be 5.7 ± 8.9° in younger healthy participants, but is significantly greater, at 17.6 ± 7.2°, in older healthy participants [4]. The LR angulation increases with aging in healthy individuals, and this increase is further pronounced in SES (22.4 ± 5.6° in DPE, 23.6 ± 13.1° in CVS) [4]. The mean LR angulation in the current study was 22°, which was approximately 4° greater than the mean LR angulation of 17.6° in older healthy participants in Chaudhuri et al.’s study [4]. In Chaudhuri et al.’s study [4], the percentage of ruptured LR-SR bands was 64% in DPE, 91% in CVS, 0% in younger controls, and 0% in older healthy participants. In this study, the percentage of ruptured LR-SR bands was 100% in SES. In the current study, the mean LR angle and percentage of LR-SR band ruptures in the patients with SES were greater than those in older healthy participants reported by Chaudhuri et al. [4], as were the patients with SES reported by Chaudhuri et al. [4].

Although healthy older adults do not show significant differences in the vertical position of the IR pulley compared to younger adults [12], the increased inferior displacement of the IR pulley in patients with SES in this study was highly correlated with the increased inferior displacement and tilt angle of the LR pulley. One reason for this could be the influence of the histological characteristics of the LR and IR pulleys. In healthy participants, the LR-SR band extends to the supraorbital side with aging [3], and the global side of the LR pulley and the orbital side of the inferior oblique (IO) pulley, where the LR-IO pulley couples, have lower collagen thickness and content [9, 16]. In patients with SES, we speculated that the same changes that cause inferior displacement of the LR owing to progressive age-related connective tissue degeneration in the LR-SR band also cause inferior displacement of the IR at the LR-IO coupling site, which has similar connective tissue characteristics as the LR-SR band and is continuous with the IR pulley (Fig. 2). In this study, the inferior displacement of the IR pulley showed the highest correlation coefficient with the inferior displacement of the LR pulley, making it the most indicative factor associated with inferior displacement of the LR pulley.

This study had a few limitations. First, this was a retrospective study with a small sample size, and both eyes of all patients were used for statistical analysis without differentiation by disease type. Additionally, it was not possible to extract the orbital MRI data of healthy older participants from medical records in the current study; therefore, a more comprehensive assessment of the conditions should be conducted with a larger sample size, by differentiating the types of diseases and adding data from healthy older participants. Second, while the positions of the RM pulleys could be compared between the patients and the control group, the tilt angles of the RMs were not available for the control group; hence, no comparison with the patient group was conducted. Therefore, the relationship between the tilt angles of the RMs and the positions of the RM pulleys requires further confirmation through comparisons in a large number of patients and healthy participants. Third, because there are no international diagnostic criteria for SES, the diagnosis in this study adhered to the diagnostic criteria outlined by Wei et al. [5]; however, cases with high myopia were excluded from the current study, and it is possible that the differences in the SES diagnostic criteria influenced the results. The criteria used for SES diagnosis differed slightly from those utilized in previous reports, such as a study that included high myopia in SES even in the absence of significant myopic degeneration [8], and a study that primarily used clinical findings to diagnose SES and excluded high myopia [1]. Therefore, caution should be exercised when comparing the results of this study with those of previous studies. Fourth, the subjects in this study included those from the period when SES was first reported [3, 4]; the diagnosis of SES was made according to the SES definition of this study, but there were some missing clinical findings and examination data in the retrospective cross-sectional case series.

In conclusion, the LR muscle angle is a valuable indicator of the inferior displacement of the LR and adjacent RM pulleys in patients with SES without high myopia. Hence, when interpreting MRI scans of patients with SES in whom uncertainty exists regarding the presence or absence of inferior LR displacement, evaluating the presence or absence of increased temporal tilt in the superior LR may prove advantageous for SES diagnosis.
